# Asiatic Cholera

**Published:** 1848-11

**Authors:** 


*Asiatic Cholera.*—The event which has been so long anticipated has at length occurred, for well-authenticated cases of cholera have been reported in London and one or two other places. The first note of alarm was sounded at Hull, where some sailors died in a few hours "with all the symptoms of the Asiatic disease." Edinburgh was shortly after seized with terror from the rport of five cases having occurred there ; and while we were looking with some degree of consternation towards the northern capital, "Great Babylon" herself was invaded by the erratic pestilence. Upon a close investigation of the

Hull cases, however, it is evident that the mortal symptoms resulted more from the violation of the hygienic rules than the direct attack of an epidemic pestilence. Beer and plums taken in immoderate quantities by individuals exposed to atmospherical vicissitudes, and called to undergo a considerable amount of bodily fatigue, will at any time set up a gastric irritation not easily allayed ; and it would be an insult to science to assert that these cases were produced by contagion or infection. Medicine has made too much progress within the last few years to be panic-stricken by such occurrences as these, for it is an attribute of ignorance to feel alarm where there are no just grounds for it. Even the first Edinburg cases have been considered of a doubtful character ; and we must, therefore, conclude that as yet only a few cases of true Asiatic cholera have occurred in our country.

To what cause, then, are we to attribute these cases ? Has the disease been imported, as we are compelled to believe, if we adopt the theory of its being contagious ? We have no proofs whatever that this is the case : and facts connected with the history of Asiatic cholera, furnished by men who have had the most extensive opportunities of studying it, prove incontestably that it is not what we commonly understand to be a contagious malady. We have here all the analogies of an epidemic,—originating in the East, extending from one country to another, till people of almost every language have felt its power. When it visited this country seventeen years ago the profession was unprepared for its attacks; it was supposed to be a malady which gave its victims no warnings of its approach, and, like the plague which once swept off its tens of thousands in this metropolis, threatened destruction to all who touched the sick. Viewed through the mists of ignorance, it assumed the most horrible forms, and men, frightened out of all propriety, adopted the most extraordinary expedients to escape its attacks. The armies of some European countries were drawn up in battle array against it; their bristling bayonets were passed as easily and as swiftly as the wind. Quarantines were established in vain; and pesthouses to which the victims were removed, offered no security to those in health by their isolated positions. Wherever the disease appeared it bade defiance to all human efforts ; and, when it seemed wearied with slaughter in one place, it passed on to another to execute there the work of death.

Since 1831 we have become better acquainted with cholera, and it is found not to be half so terrible as was supposed. Instead of making its attacks suddenly, it gives warnings of its approach ; instead of being contagious, it is simply an epidemic ; instead of resisting the judicious application of medicine, it is amenable to treatment.

There is one well ascertained fact, in reference to cholera, which is calculated to remove all improper fears, and with them the barbarous precautions which have been adopted to arrest its progress, and that is its non-contagiousness. Acting upon this doctrine, the Board of Health has not thought fit at the present time to recommend the establishment of cholera hospitals, though the Government still appears disposed to put in force, to some extent, the quarantine laws. If cholera is not contagious, then quarantine, should be dealt with in the same manner as pesthouses—done away with.

The following observations have been published by the Central Board of Health, Dublin :—

"It seems to be a well-established fact, and one that cannot be too strongly impressed upon the minds of the people generally, as upon this fact depends the best hope of successfully contending with the disease, that in nearly all cases of cholera there are two stages of the disease, the first being merely diarrhœa, or simple looseness of the bowels; the second being the stage of collapse, or blue cholera, marked by cramps, failure of the circulation, lividity of the skin, cold, clammy perspiration, and all the other well-known symptoms of the disease. In the first stage of the disease medical treatment is frequently successful; in the second stage too often of no avail.

"The first stage, diarrhœa, or mere looseness of the bowels, may be of only a few hours' duration, or may continue from one to several days. It is most important to bear in mind that this diarrhœa may be entirely without pain; indeed, it most frequently is without pain, or merely accompanied with trifling griping or uneasiness. This absence of pain, or the little accompanying uneasiness, has too often thrown the patient off his guard, who has thus neglected the warning of his danger, and has allowed the time for cure to pass by.

"It may be safely asserted that, during the prevalence of an epidemic of cholera, diarrhœa, or looseness of bowels, which is free from pain, **is more dangerous, more likely to be the first stage of the disease,** than diarrhœa accompanied with griping or pain. Let it, then, be clearly understood that, when the epidemic is prevalent, mere looseness of the bowels, with or without pain, may be the commencement or first stage of cholera—that the disease is generally curable in this stage, and that not a moment should be lost in applying for relief.

"To afford this immediate relief, the commissioners of health recommend that every existing medical institution, whether hospital or dispensary, should be open day and night during the prevalence of cholera to all applicants, without distinction, where all who apply should obtain, without a moment's delay, advice and medicine; and from which all the poor who may be unable to leave their dwellings may be visited with promptitude, and supplied with medicine at home, or transferred, if requisite, to hospital.

"To effect these objects the following arrangements are recommended :—

"1stly. The prescribing-room of every medical institution, whether hospital or dispensary, should be open day and night, without intermission, during the prevalence of cholera, and a medical officer should be in constant attendance to prescribe for all applicants.

"2dly. Each hospital and dispensary should have a certain district allotted to it, and the attending porter or clerk should keep a book in which he should enter the names and residences of all applicants for relief within the district who are unable to leave their homes.

The book should show the time of application, and the name and residence of tbe patient.

"3dly. A second medical officer should be constantly in readiness to receive the names of all such applicants, and to proceed without delay to visit them. The visiting physician, instead of writing a prescription at the residence of the patient, should be provided with a small portable pocket medicine-box, containing the medicines most generally required, made up in such a form as to render their administration as speedy and as simple as possible. Such portable medicine-boxes can be procured at a very small cost, or may be made up, on an emergency, of pasteboard, or thin board, or tin, in the form of a book about seven inches long, four inches broad, and one inch deep, with one of the sides to fold back or open on hinges. The services of an apothecary will be required to keep up a constant supply of the medicines required, made up ready for use. The medical officers will generally give formulæ for the medicines they deem best. The following may, however, serve as an example of what should be generally provided in the pocket medicine-boxes. The most portable forms for the medicines are selected; the directions should, as far as practicable, be printed.

"Powders.—Carbonate of ammonia, in waxed papers, each paper containing forty grains, with the following printed directions on the outside:—'Dissolve this powder in a half pint of water; give two tablespoonfuls every hour.'

"Powders.—Compound powder of chalk with opium (pulv. cretæ c. opio), in packets each containing six papers, each paper containing ten grains of the powder, with printed directions:—'One powder every half hour until the looseness ceases.'

"Pills of powdered opium, each containing one quarter of a grain of opium, and two grains of powdered ginger, made up with the oil of peppermint. The pills to be in boxes, each box containing six pills, with a printed label:—'Opium pills, one every half hour until the looseness ceases.'

"Pills of mercury and opium, each containing one quarter of a grain of calomel, two grains of hydrargyrum c. creta (mercury with chalk), and a quarter of a grain of opium, made up with oil of caraway (which will serve to distinguish them from the plain opium pills), in boxes, each containing six pills, with a printed label:—'Mercury and opium pills, one every half hour.'

"Bottles (one or two ounce phials, with cork stoppers).

1. Containing tincture of opium (laudanum).
2. " Hoffman's liquor.
3. " tincture of rhatany.
4. " creasote.

Along with the box should be carried a small jar of strong brown mustard.

"The visiting physician should also be furnished with printed forms, for the removal to hospital of patients who are destitute of assistance in their own dwellings; in short, every measure should be adopted that will obviate the least delay. It may be necessary in some instances to establish temporary district dispensaries ; but it is most desirable, for reasons already given, that the permanent institutions should be first made available.

"It is not within the purpose of a communication such as this to go into details of treatment. There are, however, two points on which the commissioners of health feel it will not be out of place to give an opinion, viz., the employment of frictions and the allowance of drinks to the sick. The commissioners cannot recommend that fluid applications of any kind should be employed in frictions on the body or limbs, as the cold consequent on prolonged exposure and evaporation more than counterbalances any supposed good effect from friction, which, if at all used, should be made with the warm hand without disturbing the bedclothes. The commissioners also advise that when patients suffer from thirst they should in general be permitted to drink freely, as experience shows that the denial of drink does not check vomiting, while it increases very much the suffering of the patient from the burning thirst that so often accompanies the disease."—*London Medical Times.*

